# Who to engage in HIV vaccine trial benefit-sharing negotiations? An empirical proposition of a framework

**DOI:** 10.1186/s12910-024-01058-4

**Published:** 2024-05-14

**Authors:** Godwin Pancras, Mangi Ezekiel, Erasto Mbugi, Jon F. Merz

**Affiliations:** 1 Department of Bioethics and Health Professionalism, School of Public Health and Social Sciences, Muhimbili University of Health and Allied Sciences, P.O. Box 65001, Dar es Salaam, Tanzania; 2 Department of Behavioral Sciences, School of Public Health and Social Sciences, Muhimbili University of Health and Allied Sciences, P.O. Box 65001, Dar es Salaam, Tanzania; 3 Department of Biochemistry and Molecular Biology, School of Biomedical Sciences, Muhimbili University of Health and Allied Sciences, P.O. Box 65001, Dar es Salaam, Tanzania; 4 Department of Medical Ethics & Health Policy, Perelman School of Medicine, University of Pennsylvania, Blockley Hall Floor 14, 423 Guardian Drive, Philadelphia, PA 19104-4884 USA

**Keywords:** Benefit-sharing, Framework, Engagement, Negotiations, Decision-making, HIV vaccine trials, Tanzania

## Abstract

**Background:**

A morally sound framework for benefit-sharing is crucial to minimize research exploitation for research conducted in developing countries. However, in practice, it remains uncertain which stakeholders should be involved in the decision-making process regarding benefit-sharing and what the implications might be. Therefore the study aimed to empirically propose a framework for benefit-sharing negotiations in research by taking HIV vaccine trials as a case.

**Methods:**

The study was conducted in Tanzania using a case study design and qualitative approaches. Data were collected using in-depth interviews (IDI) and focus group discussions (FGD). A total of 37 study participants were selected purposively comprising institutional review board (IRB) members, researchers, community advisory board (CAB) members, a policymaker, and HIV/AIDS advocates. Deductive and inductive thematic analysis approaches were deployed to analyze collected data with the aid of MAXQDA version 20.4.0 software.

**Results:**

The findings indicate a triangular relationship between the research community, researched community and intermediaries. However, the relationship ought to take into consideration the timing of negotiations, the level of understanding between parties and the phase of the clinical trial. The proposed framework operationalize partnership interactions in community-based participatory research.

**Conclusion:**

In the context of this study, the suggested framework incorporates the research community, the community being researched, and intermediary parties. The framework would guarantee well-informed and inclusive decision-making regarding benefit-sharing in HIV vaccine trials and other health-related research conducted in resource-limited settings.

**Supplementary Information:**

The online version contains supplementary material available at 10.1186/s12910-024-01058-4.

## Introduction

Benefit-sharing has gradually become a focus of ethical inquiry for studies conducted in developing countries [1]. In developing countries, most individuals and communities that participate in research are vulnerable [2, 3]. Benefit-sharing helps to achieve the ethical goals of justice and prevent the exploitation of such communities. Some widely referenced frameworks that could be used to guide benefit sharing include reasonable availability, fair benefits, and human development frameworks [4–7]. The reasonable availability framework emphasizes the need to ensure potential benefits directly arising from the study are reasonably available to host communities and individual participants. The fair benefits framework goes beyond the potential end products of research to include other benefits or advantages accrued by implementing a study in a target locale. Finally, the human development frameworkfocuses on the pre-existing needs of the researched community to define the benefits that will most meet those needs. However, the aforementioned frameworks offer limited guidance on benefit-sharing negotiation and decision-making. In the absence of a fair decision-making process, benefit-sharing plans might be unjust.

Effective communication and engagement of all parties, whether or not they are directly affected by the research and its outcomes, are crucial factors to be considered in the planning, negotiation, and decision-making processes of research [8]. The negotiation approach in decision-making involves “efforts to adjust to the preferences and expectations of bargaining peers in which concessions are exchanged according to the principle of reciprocity” [9]. For that reason, the methods developed for community-based participatory research (CBPR) and “community-engaged research” (CEnR) strive to engage a broad constituency of stakeholders in the decision-making processes as equal partners [10, 11]. Decisions are not made for but with the researched community despite the contextual or structural vulnerabilities of the research environment [12, 13]. This includes making decisions about sharing potential research benefits with participants and communities. As noted by Wendler and Shah, “the primary challenge will be to determine *who* should be involved in deciding what benefits are provided to the host community” [14]. But CBPR and CEnR methods provide little guidance on whom and when to engage multiple stakeholders [15]. Moreover, the extent to which researched communities ought to be engaged and the responsibilities of different partners in negotiations and decision-making is less explicit.

Therefore, this study presents empirical evidence of whom and when to engage different parties in benefit-sharing decisions, focused on HIV vaccine trials conducted in Tanzania. From 2007 to the present time, 8 HIV vaccine trials have been conducted in Tanzania in two locations: Dar es Salaam and Mbeya region [16–21]. The trials have been limited to phase 1/2a and recruited healthy adults and youth. This study findings hold the potential to empirically inform and improve frameworks of benefit sharing especially for trials conducted in resource-constrained settings.

## Methods

The study was conducted as part of PhD project on ethical implications for sharing HIV vaccine trial benefits in Tanzania. The methods used in this study have been documented elsewhere [22, 23]. Briefly, the study was conducted in Dar es Salaam and Mbeya region in Tanzania. A qualitative case study design involving in-depth face-to-face interviews (IDIs) and focus group discussions (FGDs) was employed. The IDI and FGD interview guides have been attached as supplementary file. The study population was purposively selected and included researchers or principal investigators of HIV vaccine trials, Institutional Review Board (IRB) members, representatives of HIV advocacy groups, a policymaker and community advisory board (CAB) members in Tanzania. That is, the selected study participants had either experience or lived within HIV vaccine trial host communities in Dar es Salaam and Mbeya. To obtain a shared understanding of benefit-sharing, FGDs were conducted only with CAB members. In total, three FGDs, each with 5 participants, were conducted. Regarding data analysis, the thematic analysis method was used in which the coding process applied both deductive and inductive techniques to develop the themes. The process was aided by MAXQDA version 20.4.0 which is computer-assisted qualitative data analysis software. All study participants provided written informed consent. International and local research ethics guidelines were followed during the conduct of this study [24, 25].

## Results

### Characteristics of the interviewees

The study included a total of 22 individual IDIs and 15 focus group participants with an age range between 21 and 80 years old. Females made up 14 out of 37 study participants. The majority of the study participants (20 out of 37) had attained university education. On average, the work experience of researchers, IRB members and CAB members was 20, 12 and 7 years respectively.

### Description of findings

#### Parties to engage in benefit-sharing negotiations

Interviewees believed that the negotiation of benefits should engage various key players including researchers, community advisory boards (CAB), research regulatory institutions and the government among others. These groups can broadly be categorized as the research community, researched community and intermediaries. One of the interviewees hinted that “whatever model is adopted it is important not to leave behind any partner” (IDI: HIV Advocacy #7).

#### Research community

**Fig. 1:**
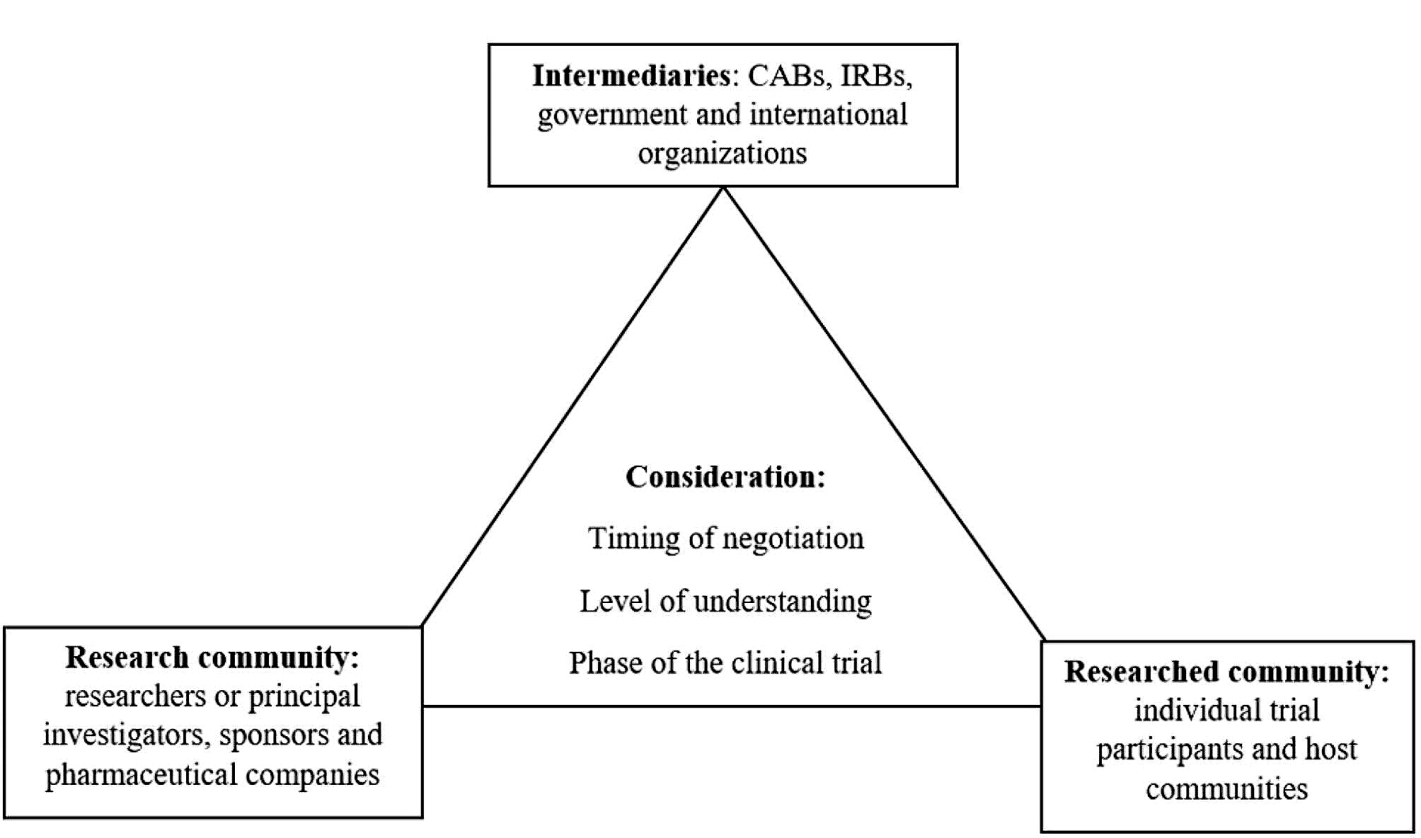
The stakeholders benefit-sharing engagement framework

The research communities comprise researchers or principal investigators, sponsors and pharmaceutical companies as indicated in Fig. 1. For researchers, interviewees believed that their engagement is important since they are the ones who initiate and implement studies. The researchers should be proactive from the start in recognizing the potential benefits that can arise from their research. This was noted by one of the FGD participants that “…the first people to identify benefits or harms are the researchers who started the trial” (FGD2: Participant #3). A similar perception was extended to sponsors including research host institutions. Thus, research goals and benefits would normally align with that of the sponsor as exemplified by one of the interviewed researchers that “training activities [within the institution] were aligned with the HIV vaccine trial activities…and are within the local university research and development priorities” (IDI: Researcher #5).

Additionally, one of the interviewees reported that during the submission of research proposals for funding by international organizations, one of the critically examined components is the access of intervention to the population. He noted that “…reviewers of the submitted grant applications should look at the proposed interventions and see if it would be accessible to the poor and it is acceptable” (IDI: HIV advocate #7). This requirement is cemented by funders entering into a contractual agreement with researchers to make sure that potential benefits would be shared. However, after the trials are completed it was difficult for funders to do the follow-up: “…once the project is finished it is very difficult to do follow-up… we leave it in the hands of the sponsors and trial team” (IDI: HIV advocate #7). Concerning pharmaceutical companies, their importance is based on the manufacturing of the vaccine that would be distributed to the public. Study participants felt that it is the manufacturing process that would define the price at which the vaccine will be made available. Some participants noted that recently big pharmaceutical companies have become less interested in HIV vaccine development due to less expected benefits.

#### Researched community

From the participants’ views, the researched community included individual trial participants and communities being researched (see Fig. 1). Apart from trial participants being directly burdened by the trial, they are also well-informed compared to other members of the community. Interviewees thought that they should not be left out of the decision-making process. One of the interviewees reported that “those who are participating in the trial are more likely to be better informed and can convince their communities of the kind of things that are needed” (IDI: Researcher #1). However, against this view, other interviewees noted that it was wrong to assume that the community is lay henceforth it should not be engaged in benefit-sharing decisions. But again decisions by individual participants “may be influenced by some other very personal issues not shared by the community” (IDI: Researchers #2). In support of that, one of the participants who is an IRB member noted that “Participants are part of the community, if the community benefits so do the participants” (IDI: IRB member #3).

#### Intermediaries

Based on interviewees’ accounts, this included groups of people who would mediate the discussions and decisions on how to share the benefits of an HIV vaccine trial. This included CABs, IRB, government and international organizations as seen in Fig. 1. The CAB is a group made up of community representatives from which the trial is being conducted. The day-to-day interaction of the CAB with researchers and trial participants made the group more conversant with both parties. Thus researchers had to go through the CAB before they could start recruiting trial participants, one interviewee said that “the researcher cannot go straight to [recruit] the participants until the CAB agrees” (FGD 3: Participant #4). Moreover, interviewees noted that researchers, IRBs and institutions are not as independent as CABs as it was reported by one IRB participant that “CABs are free from any conflicts of interests…most of the IRBs are within institutions so they could not appropriately represent the participants” (IDI: IRB member #4). Also, the interviewees noted that not every study will have a CAB and this weakens the negotiation process “…most researches do not have CABs and to the extent, this weakens independent negotiations” (IDI: IRB member #4).

Besides, some interviewees believed that benefit negotiations should still engage research regulatory institutions such as IRB because they are the ones who approve studies to be conducted in the community. The government on the other hand was believed to be a key player in ensuring benefits reach the intended population and this included the creation of policies as noted by one of the interviewees “…country governments should take the responsibilities of ensuring that policies are made and implemented for populations in need to reap the benefits” (IDI: Researcher #5). Interviewees specifically named the Ministry of Health as a responsible arm of the government that could oversee the benefit-sharing negotiations including the logistics. Apart from the government, international organizations were also identified as important parties to be engaged in the negotiations. Interviewees believed that these organizations can negotiate with both the manufacturers and country governments when it comes to procuring HIV vaccines.

### Considerations for engagement in benefit-sharing negotiations

Interviewees noted three issues that should be considered when engaging the host community in benefit-sharing negotiations and decision-making: timing of the negotiations, level of understanding among negotiating parties, and the phase of the trial.

Concerning the timing of the negotiations, interviewees differed on whether benefit-sharing negotiations and decisions should be made before the vaccine trial is undertaken or after it is completed. Those who preferred engaging in negotiations before the trial begins said it should either be during protocol development, the IRB review meeting or consenting. Pointing to the importance of this, one interviewee noted that it would “enable you to decide on whether or not to participate in the study” (IDI: Researcher #2). Another interviewee who is an IRB member worried that “what if after the trial ends, they close their offices in Tanzania and leave, where will you get them?” (IDI: IRB #2). The belief that prior negotiations of benefits with potential participants would influence their decision to participate was refuted by one of the interviewees who narrated that “the importance of knowing the benefits first does not make him want to participate in the research but it gives him the freedom to choose” (FGD 3: Participant #4). On the other hand, one of the CAB members believed that the right moment to engage in discussions about sharing benefits should be after trial completion thus “after the trial is carried out in the community and the vaccine is available, it is the right time to start discussing its benefits.” (FGD 2: Participant #2). Still, some interviewees believed that benefit negotiations should be a continuous process that is, before, during and after the trial is completed.

In addition, participants noted that engaging multiple stakeholders with varied levels of understanding especially in the language of communication would pose a challenge. One of the participants who is a CAB member said that “to have an in-depth conversation, it is very important that the conversation is compatible with the level of understanding of those you are talking to” (FGD 1: Participant #1). In addition, deciding whom to engage in benefit-sharing decisions depended on the phase of the trial, that is, a wide array of stakeholders would need to be engaged as trials move on to late phases. One participant who is an IRB member noted that “as clinical trials move on to higher phases the negotiations should include a wider array of stakeholders in the trial host community” (IDI: IRB #4).

## Discussion

The research community, intermediaries and researched communities are key stakeholders worth being engaged in benefit-sharing negotiations. The engagement to be fruitful it ought to consider the timing of the negotiations thus before, during or after the trial has ended. But also the discrepancies in the level of understanding of negotiating parties and the phase of the clinical trial from which the benefits are expected.

The research community derives the initiation, implementation and development of the product. For that reason, the research community extends beyond the researchers to encompass sponsors and pharmaceutical companies. Ethical guidelines have called on researchers and sponsors to ensure that the product being researched or any other advantages are enjoyed by researched communities. For example, the guidelines issued by the Council for International Organization of Medical Sciences (CIOMS) state that “as part of their obligation, sponsors, and researchers must also make every effort, in cooperation with the government and other relevant stakeholders, to make available as soon as possible any intervention or product developed…for the population or community in which the research is carried out” [25]. Thus the term ‘cooperation’ may well translate to the engagement of diverse stakeholders to make informed benefit-sharing decisions. Simply, researchers, sponsors and pharmaceutical companies cannot decide in isolation about what, when and how benefits ought to be shared. A similar conception was held by the interviewed study participants. Pharmaceutical companies would play a big role in commercial clinical trials intending to develop a commercial product, the HIV vaccine could be far from being one.

Furthermore, research sponsors have been found to have a crucial role when it comes to sharing of benefits of HIV vaccine trials. So far the word ‘sponsor’ in research is defined and understood differently [26]. But in developing countries, it is commonly used when referring to academic and research institutions that obtained funding from another institution or organization usually from the global north to carry out a clinical trial or any other research. Besides the institution that hosts the local principal investigator or researcher is usually designated as the sponsor. The implication of this is that the sponsor will have to consider how the institution will benefit by hosting the trial and at the same time think about the benefits of researched communities. As reported by participants, sponsors would usually align benefits to the institutional existing priorities. However, it is very unlikely it would be the same for trial participants and hosting communities whose priorities may be unknown to the sponsors or extend beyond what can be offered by the study. The implication of which, one side would seem to be reaping more benefits at the expense of the other. Engaging other stakeholders, the researched community and intermediaries who be a probable solution.

For the researched community, it is the one that carries the burden of the trial from which they expect to benefit. Otherwise, it is an injustice and undermines the principle of reciprocity [27]. HIV vaccine trial participants and host communities expect some benefits by virtual of their participation [23]. However, as noted by Neema Sofaer, reciprocating benefits largely depends on “who should reciprocate and to whom?” [27]. According to interviewees, it is the research community and intermediaries that bear the obligation to reciprocate the benefits to the researched community. And the researched community extends beyond individual participants to include non-participants like the host community. When it comes to who to engage, the two parties, individual participants and the host community are not mutually exclusive. Thus one party cannot take one’s position in making trial benefit decisions. This implicates the sense of communalism or Ubuntu in African countries like Tanzania where community decisions would override that of the individual [28]. The rationale is that what the community decides as a benefit would eventually benefit the individual participants. Otherwise, the decisions made by individuals may not reflect that of the community. But whether the individual participant identifies as part of the community or not risks of who to engage in decision-making should be mitigated [11]. So to navigate between these competing claims, an important player who understands the researched and research community would be warranted, the intermediaries.

For this study, intermediaries included CAB members, IRB members, the government and international organizations. Intermediaries would largely mediate the discussion and decisions regarding benefit-sharing lather becoming ultimate decision-makers. Similar to other clinical trials, CAB in HIV vaccine trials, are made up of representatives from the community and usually act to advance the goals of the research community while protecting the interest of the researched community [29]. However, not all clinical trials will have a fully functioning CAB and this could create a loophole when deciding about benefits to be shared. The IRB, government and international organizations cannot replace its role because CAB members are from the grassroots where the trial is or has been conducted. More importantly, CABs may experience less conflicts of interest compared to IRBs that are usually housed under research (sponsor) institutions. Still, the risk of conflict of interest among CABs cannot be ruled out since most are built and logistically supported by the research community [11].

The IRB engagement in benefit-sharing decisions cannot be underestimated. In essence, IRBs are mandated to ensure the well-being of the study participants through protocol reviews and oversights. Given its experience in ethics, the IRB would be able to identify and advise about moral issues at stake in the benefit-sharing process. Engaging the government and political leaders in research and benefit-sharing decisions is paramount. Governments bear the responsibility to ensure the well-being of their people. More importantly, the government through its ministries would be responsible for the logistical distribution of the vaccines or products developed. However, for countries with limited resources and a plethora of demanding needs country government would need a helping hand. At this juncture, this calls for international organizations that would help facilitate the logistics of the developed product, between the government, research community and the researched community. Mechanisms already exist to form global public/private partnerships (PPP) that would enable vaccines to be shared with researched communities [30]. The recent COVID-19 outbreak has evidenced governments and international organizations forming unprecedented coordination to ensure equitable access to COVID-19 vaccines in poor countries including Tanzania [31]. Moreover, engaging international organizations in benefit-sharing decisions would ensure that the values of global solidarity are upheld [32]. Thus, benefits which arise from the trial are not selfishly limited to participating countries or communities.

Despite the call for this triangular engagement between research, intermediaries and researched communities, three issues ought to be considered: the timing of the negotiations between parties, the level of understanding and the phase of a clinical trial. For timing, some ethics guidelines recommend benefit negotiations before and during the conduct of the study [25, 33]. Morally, this ensures that each stakeholder’s values and preferences are taken into account in deciding the potential benefits. However, this could compromise altruistic intentions and raise a sense of undue influence to research participation when negotiators turn to deception tactics to preserve their interests [34]. Making false promises and fabrication are some of the common deceptive tactics that could be employed in negotiations [35]. On the other hand, negotiations become hinged on actual rather than potential benefits after the study is completed. This provides an opportunity for negotiators to negotiate based on stakeholders’ actual contribution in realizing the study benefits [23]. Deciding whether negotiations should be held before, during or after could depend on the weight that negotiating parties assign to each of the outlined strength.

Regarding the level of understanding, the language used as a medium of communication could affect the informed engagement of the researched community. But also expected benefits of a phase 1 clinical trial would be different from that of a phase 3 clinical trial and that warrants a difference in who to engage in deciding the benefits. We call on studies to further explore and assess these concepts as applied not only in HIV vaccine trials but also to other types of community-based research. A grounded theory approach would be well suited. In addition, it will be of essence to explore the question of who oversees the engagement process.

The study limitation includes the incapacity to statistically generalize the study findings to other settings. However, the use of an instrumental case-study design allows the findings of this study to be analytically generalizable to other similar settings or trials [36]. Moreover, the study deployed triangulation in terms of: the study population (researchers, CAB members, HIV advocacy members, a policymaker and IRB members); study area: two administrative regions (Dar es Salaam and Mbeya); and data collection methods: IDIs and FGDs.

## Conclusion

Concepts like ‘engage with’, ‘consult with’ and ‘cooperate with’ are primarily used in research ethics guidelines to indicate the need for a ‘plan’ for benefit-sharing among competing research stakeholders. This study empirically indicates a triangular relationship between the research community, researched community and the intermediaries in benefit-sharing negotiations. However, three considerations ought to be taken into account when utilizing the proposed empirical framework: the timing of the negotiations, the level of understanding between parties and the phase of the clinical trial. The proposed framework offers an informed and inclusive approach towards sharing the benefits of clinical trials in resource-constrained settings. Moreover, the framework augments other existing frameworks for sharing the benefits by explicitly stating the key stakeholders to engage in benefit negotiations. We call on scholars, researchers, regulatory institutions and the scientific community to adapt and improve the proposed framework.

### Electronic supplementary material

Below is the link to the electronic supplementary material.

Supplementary Material 1

## Data Availability

The datasets used and/or analyzed during the current study are available from the corresponding author on reasonable request.

## Abbreviations

CABs: Community Advisory Boards
FGDs: Focus Group Discussions
IDI: In–depth Interviews
HIV: Human immunodeficiency virus
