# Supplementary material for: Who to engage in HIV vaccine trial benefit-sharing negotiations? An empirical proposition of a framework
Source: BMC Med Ethics. 2024 May 14;25:54. doi: 10.1186/s12910-024-01058-4 (PMC11092097; doi:10.1186/s12910-024-01058-4)
Supplement: Supplementary file 1 — Supplementary Material 1 [file 12910_2024_1058_MOESM1_ESM.docx]

## Interview guides

## Appendix 1: Interview Guide for Researchers; English Version

**Introduction:** Thank you very much for agreeing to participate in this interview. You have been selected among other members because you have experience and knowledgeable in HIV vaccines studies. During this interview there are no wrong answers, all answers are accepted so kindly feel free. I would like to remind you that this interview may take 30 to 60 minutes and it will be audio recorded to grasp points that could be difficult to write down but also for easy analysis of the information later on.

**Qn1**. I would like us to start by introducing yourself:-

| a | Gender |  |
| --- | --- | --- |
| b | Age |  |
| c | Work experience (years) as clinical trial scientist |  |
| d | Profession |  |
| e | Major responsibility(ies) in an HIV vaccine trial |  |
| f | Number of HIV vaccine trials involved |  |

**Qn2.** **What do you think are the benefits for participating in an HIV vaccine trial (to you/ participants)?**

SUB QUESTIONS:

1. What benefits should come directly from the HIV vaccine trial?
2. Should benefits of the HIV vaccine trial be given freely, subsidized or at market price? Why?
3. What would you consider first as a researcher: the ‘amount of benefits’ or the ‘type of benefits’ participants receive from participating in HIV vaccine study? Why?
4. Do you think benefits should only increase with the increase in risks associated with the HIV vaccine trial? Why?
5. How has an HIV vaccine trial you participated contributed/ should contribute to capacity building?
6. How did/should capacity building activities reflect the society needs?

**Qn3**. Who were the beneficiaries of your HIV vaccine trial? Why?

SUB QUESTIONS:

1. Were benefits of the trial you participated shared with other people or groups which did not participate in study? Why?
2. Were benefits of an HIV vaccine trial shared with the community from which the study took place? Why?
3. Should other nations benefit from an HIV vaccine trial you participated/ currently participating? Why? But do you think your nation should benefit first?

**Qn4**. How have benefits been shared/ ought to be shared in an HIV vaccine trial you participated?

SUB QUESTIONS:

1. What would make someone receive benefits from an HIV vaccine trial rather than the other?
2. Do you think the benefits of HIV vaccine trial should be equally shared? Why?
3. When sharing the benefits of your HIV vaccine trial with other people or groups, should you consider their level of contribution to the study? Please tell me more about that.
4. If some participants participated honestly or contributed more to the study than the other, do you think they should be given the first consideration when sharing benefits? Why do you think so?
5. What do you think is more important when sharing the benefits of HIV vaccine trials to consider: requirements of the community or individual participants first? Why?

**Qn5.** What would be your advice regarding the sharing of benefits of HIV vaccine trials?

SUB QUESTIONS:

1. What should be done to ensure that benefits shared reach targeted population?
2. Whom do you think should take the responsibility to ensure benefits of your study are available to you or your community? Why?
3. Whom do you think the benefits of an HIV vaccine trial should be negotiated with? Why?
4. When should it be the appropriate time to negotiate benefits: before or after the HIV vaccine trial begins? Why?

**Closure of the interview**: Is there anything else that you would like to share with me about what we have just discussed?

Thanks!

## Appendix 2: Interview Guide for members of HIV Advocacy groups and Policymakers; English Version

**Introduction:** Thank you very much for agreeing to participate in this interview. You have been selected among other members because you have experience and knowledgeable in HIV vaccines studies. During this interview there are no wrong answers, all answers are accepted so kindly feel free. I would like to remind you that this interview may take 30 to 60 minutes and it will be audio recorded to grasp points that could be difficult to write down but also for easy analysis of the information later on.

**Demographic information**

(*Filled on a separate table*)

Qn1. How far have you (or your institution) been involved in HIV vaccine trials?

SUB QUESTION

1. And what is your opinion regarding HIV vaccine trials in Tanzania
2. What do you think are the benefits for participating in an HIV vaccine trial
3. In case we get an HIV vaccine, do you think they should be given freely, subsidized or available at market price?

Qn2. Should benefits be shared with other countries/ communities that did not participate in the clinical trial? Why?

1. Do countries holding HIV vaccine trials have obligations to share benefits with other countries? Why do you think so?

Qn5**.** What would be your advice regarding the sharing of benefits in HIV vaccine trials?

SUB QUESTIONS:

1. What should be done to ensure benefits shared reach targeted population?
2. Whom do you think should take the responsibility to ensure that, benefits of HIV vaccine trials are fair and available to the community?
3. Whom do you think benefits of an HIV vaccine trial should be negotiated with? Why?
4. When do you think is the appropriate time to negotiate benefits: before or after the HIV vaccine trial begins? Why?

**Closure of the interview**: Is there anything else that you would like to share with me about what we have just discussed?

Thanks!

## Appendix 3: Interview Guide for IRB members; English Version

**Introduction:** Thank you very much for agreeing to participate in this interview. You have been selected among other members because you have experience and knowledgeable in reviewing HIV vaccines studies. During this interview there are no wrong answers, all answers are accepted so kindly feel free. I would like to remind you that this interview may take 30 to 60 minutes and it will be audio recorded to grasp points that could be difficult to write down but also for easy analysis of the information later on.

**Qn1**. I would like us to start by introducing yourself,

|  | Gender |  |
| --- | --- | --- |
| b | Age |  |
| c | Work experience (years) as an IRB member |  |
| d | Profession |  |
| e | Number of HIV vaccine/clinical trials reviewed |  |

**Qn2.** **What do you think are the benefits for participating in an HIV vaccine trial?**

SUB QUESTIONS:

1. Which benefits could come direct from the HIV vaccine trial(s) you reviewed? Do you think they should be given freely, subsidized or at market price
2. As a reviewer, what would bother you: the ‘amount of benefits' or the ‘type of benefit' participants receive in an HIV vaccine trial? Why?
3. Do you think benefits should only increase with the increase in risks associated with the HIV vaccine trial? Why?
4. Were there any capacity building activities indicated in an HIV vaccine trial protocol you reviewed? Please tell me more about that
5. How do you ensure capacity building activities reflect the society needs?

**Qn3**. Who were/should be the beneficiaries of most HIV vaccine trials you reviewed? Why?

SUB QUESTIONS:

1. What criteria should be followed in determining recipients of benefits from an HIV study?
2. Should benefits of HIV vaccine trials be shared with the community hosting the trial? Why?
3. Should other nations benefit from an HIV vaccine trials undertaken in Tanzaia? Why? But do you think your nation should benefit first?

**Qn4**. How were/ should benefits be shared in an HIV vaccine trial you recently reviewed?

SUB QUESTIONS:

1. In most HIV vaccine trials you have reviewed, where/should the resulting benefits be shared equally? Why?
2. If some participants participated honestly or contributed more to the study than the other, do you think they should be given the first consideration when sharing benefits? Why do you think so?
3. When reviewing benefits of an HIV vaccine trial, what do you consider as most important: requirements of the community or individual participants? And Why?

**Qn5.** What would be your advice regarding the sharing of benefits in HIV vaccine trials?

SUB QUESTIONS:

1. What should be done to ensure benefits shared reach targeted population?
2. Whom do you think should take the responsibility to ensure that, benefits of HIV vaccine trials are fair and available to the community or trial participants? How?
3. Whom do you think benefits of an HIV vaccine trial should be negotiated with? Why?
4. When do you think is the appropriate time to negotiate benefits: before or after the HIV vaccine trial begins? Why?

**Closure of the interview**: Is there anything else that you would like to share with me about what we have just discussed?

Thanks!

**Appendix 4: Focus Group Discussion guide for Community Community Advisory Board members [English Version]**

**Introduction:** Thank you very much for agreeing to participate in this discussion. You have been selected as representative members of the community because you have experienced and harbored an HIV vaccine trial(s) in your village/town/street/hamlet. During our discussions, there are no wrong answers; all answers are accepted so kindly feel free. I would like to remind you that our discussion may take 1 to 2 hours and it will be audio recorded to grasp points that could be difficult to write down but also for easy analysis of the information later on. Please do use the numbers provided to identify yourself or address another member during our discussion.

**Qn1**. I would like us to start by everyone introducing him/her self.

When was your CAB established? ………..

How often do you meet? …………

***A simple registration form with serial number for each individual will be used to collect information on their age, marital status, education level, length of time as a board member (or resident of the village/hamlet/town)(It will be prepared at the field depending on the number of participants)***

**Qn2**. Generally, what do you think are the benefits for participating in an HIV vaccine trial?

SUB QUESTIONS:

1. What benefits come/ should come direct from that HIV vaccine trial you participated as community representative? Where benefits given freely, subsidized or sold to you at market price and why?
2. What bothers you as a community: the ‘how much of benefits’ or the ‘type of benefit’ you get from participating in an HIV vaccine trial? Why?
3. How did/ should an HIV vaccine trial you participated contribute to capacity building activities in your community?

**Qn3**. Who were the/should be the beneficiaries of an HIV vaccine trial in your community? Why?

SUB QUESTIONS:

1. Do you think benefits should be shared with people only who participated in the study? Why do you think so?
2. Do you think your community should benefit from the study you previously (currently) participated? Why
3. Should other nations benefit from an HIV vaccine trial you participated/ currently participating? Why? But do you think your nation should benefit first?

**Qn4**. How have benefits been shared/ should be shared in an HIV vaccine trial your community participated/currently participating?

SUB QUESTIONS:

1. In your community who were the first people to receive the benefits of the HIV vaccine trial? Why?
2. Do you think the benefits of HIV vaccine trials were (or should be) equally shared? Why?
3. Do you think benefits of an HIV vaccine trial should be shared with your community according to your level of contribution to the study? (i.e. Communities which sacrifice more of their resources should be given more benefits compared to other communities which did not) Why?
4. What do you think is more important when sharing the benefits of HIV vaccine trials to consider: society requirements or yours first? Why so?

**Qn5.** What would be your advice regarding the sharing of benefits in HIV vaccine trials?

SUB QUESTIONS**:**

1. What should be done to ensure benefits shared reach targeted population?
2. Whom do you think should take the responsibility to ensure that, benefits of HIV vaccine trials are fair and available to the community or trial participants? How?
3. Whom do you think benefits of an HIV vaccine trial should be negotiated with? Why?
4. When do you think is the appropriate time to negotiate benefits: before or after the HIV vaccine trial begins? Why?

**Closure of the interview**: Is there anything else that you would like to share with me about what we have just discussed?

Thanks!
